# Mll5 Is Required for Normal Spermatogenesis

**DOI:** 10.1371/journal.pone.0027127

**Published:** 2011-11-01

**Authors:** Damian B. Yap, David C. Walker, Leah M. Prentice, Steven McKinney, Gulisa Turashvili, Katrin Mooslehner-Allen, Teresa Ruiz de Algara, John Fee, Xavier d'Anglemont de Tassigny, William H. Colledge, Samuel Aparicio

**Affiliations:** 1 Department of Molecular Oncology, British Columbia Cancer Research Centre, Vancouver, British Columbia, Canada; 2 Department of Pathology and Laboratory Medicine, University of British Columbia, Vancouver, British Columbia, Canada; 3 Department of Paediatrics, Addenbrooke's Hospital, Cambridge, United Kingdom; 4 Physiological Laboratory, Department of Physiology, Development and Neuroscience, University of Cambridge, Cambridge, United Kingdom; CNRS, France

## Abstract

**Background:**

Mll5 is currently a member of the Mll family of SET domain histone methyltransferase proteins but studies have also showed that it could be part of the SET3 branch of proteins. Recently, constitutive knock out animal studies have shown that Mll5 is required for proper haematopoietic stem cell differentiation, and loss of Mll5 results in synthetic lethality for genome de-methylation. Mll5 deficient male mice are infertile and here we analyse the consequences of Mll5 deficiency for spermatogenesis.

**Methodology/Principal Findings:**

Mll5 deficient male mice, but not female mice, are infertile. Here we show using RNA in-situ hybridization that *Mll5* is expressed in the germ cells of the testes of wild type mice. Consistent with the expression of *Mll5*, we demonstrate by electron microscopy, video microscopy and *in vitro* fertilisation techniques that Mll5 deficient mice have defects in terminal maturation and packaging of sperm. The defects seen include detachment of the acrosomal cap and impaired excess cytoplasm removal. Functional tests of sperm motility show a lack of progressive motility of spermatozoa from Mll5 deficient animals. None of these defects could be rescued by *in vitro* fertilization. Using microarray analysis we show that transcripts implicated in spermatogenesis are dysregulated.

**Conclusions/Significance:**

Our data demonstrate a clear role of Mll5 in mammalian spermatogenesis at the level of terminal differentiation providing further support for its classification in the SET3 branch of proteins. Moreover, this study identifies *Tlk2*, *Utx*, *Gpr64, Sult4a1, Rap2ip, Vstm2* and *HoxA10* as possible Mll5 targets that together may account for the observed spermatozoa maturation defects.

## Introduction

Spermatogenesis occurs in most male mammals throughout their lifetime. Successful self-renewal of the spermatogonial stem cells underpins this process [1], which comprises three distinct phases [2]. The first involves the mitotic proliferation of the germs cells, spermatogonia and differentiation into primary spermatocytes, which then proceed into meiosis, the second phase, to form haploid spermatids. The round haploid spermatids then elongate, condense their chromatin by replacing histones with protamines [3], develop an acrosomal cap, a tail assembly packed with mitochondria, and shed excess cytoplasm, all in the third and final phase of differentiation, termed spermiogenesis [4]. This phase culminates in the release of spermatozoa into the lumen of the seminiferous tubules. As a by-product of spermiogenesis, residual bodies containing excess cytoplasm from late stage spermatids are released into the lumen of the seminiferous tubules. These must be removed by phagocytosis by the Sertoli cells [5]. Spermatozoa are then drained from the seminiferous tubules into the epididymides and seminal vesicles for storage.

It has been reported that independently generated knockout mouse models of *Mll5* have a post meiotic spermatogenic phenotype [6]–[7]. The mammalian Mll proteins (Mll1-5, from original identification in mixed lineage leukemias) are structurally and functionally homologous to the *Drosophila* Trithorax proteins [8] and all contain a plant homeodomain (PHD) zinc finger motif and a conserved Su(var)3,9, enhancer of zest, Trithorax (SET) domain. Structural and biochemical analysis of SET domains have revealed their histone methyltransferase function associated with histone H3 Lys-4 (K4) methylation [9]; [10], and PHD fingers have been shown to act as recognition motifs for histone modifications [11].

Mll5 (KMT2E) was initially assigned to this family in part due to the sequence similarity of its PHD and SET domains to those of Mll. However, recent studies suggest that both human MLL5 and mouse Mll5, and the murine paralog, Setd5, have SET domains that are closer in sequence to the yeast SET3 and SET4 proteins [12]; [13]. Until recently, intensive attempts to detect the biochemical activity of yeast SET3 or mammalian Mll5 had failed. This may be due to the fact that glcNAcylation of mammalian Mll5 is required to confer H3K4 methylation activity [10]. Emerging experimental evidence suggests that Mll5 may be the functional homolog of the *S. cerevisiae* SET3; Mll5 was found to be part of the NCOR complex which is believed to be functionally similar to the SET3C complex [14]–[15] and siRNA knock down of several components (mammalian homologs of individual members of the yeast SET3 complex) in human cells phenocopies knock down of *MLL5*, consistent with the hypothesis that they may be part of the same complex [16].

Recently, our group and two others have described mouse knockout models for *Mll5*. Mll5 appears not be essential for embryonic development [6] [7]; [17], although reduced viability was reported and all three models show hematopoietic defects. Initial phenotypic characterisation of these models focussed on these hematopoietic defects.

Gametogenesis has not been examined in detail in the *Mll5* knockout mice, however male infertility was noted in two of the initial reports [6]; [7]. Interestingly, in yeast, the deletion of *set3 (Δset3)*, resulted in normal vegetative growth and development [18], but reduced viable ascus formation caused by the deregulation of sporulation genes during meiosis but not during vegetative growth [18]. The molecular mechanism(s) by which SET3 regulates genes involved in yeast gametogenesis is not known. However, it is conceivable that it interacts with nucleosomes via its PHD domain [11], bringing with it a protein complex containing histone modifying enzymes which exert their activity and regulate gene expression involved in gametogenesis [18].

In this report we show using electron microscopy, video microscopy and *in vitro* fertilisation techniques that male fertility is impaired in *Mll5^tm1Apa^* mice, due to multiple defects in terminal spermatozoa differentiation / maturation. Thus providing further experimental evidence supporting that idea that Mll5 is that functional homolog of yeast SET3. We also show specific deregulation of several important gene transcripts in the testis, which may be putative targets of Mll5 regulation.

## Results

### Homozygous *Mll5^tm1Apa^* male mice are infertile

The generation of a loss of function allele for *Mll5* is described in detail elsewhere [6]. Briefly, we generated *Mll5^tm1Apa^* by insertion of a β-galactosidase (β-Gal) reporter cassette and neomycin resistance cassette under an independent promoter (MC1) in coding exon 3 of the murine *Mll5* locus (exon 4 in ENSMUST000000094962) in 129S6 (129SvEv) embryonic stem cells resulting in the deletion of 180 bp of coding sequence. This disrupts the 5′-most coding exon generating a frame shift. This allele (with the resistance cassette in place) was passed to the germline and backcrossed twice to 129S6 wild type mice before intercrossing. The loss of full-length mature Mll5 protein was verified by Western blotting [6]. Homozygous *Mll5^tm1Apa^* male and female mice can survive through adulthood but display pleiotropic haematopoietic and maturation defects [6] [17]. Importantly, heterozygous *Mll5^tm1Apa^* intercrosses result in Mendelian ratios of homozygous, heterozygous and wild type pups at embryonic day 16.5, although non-Mendelian ratios were observed later in development, likely due to loss of homozygous pups between birth and weaning secondary to an immune defect [6].

No *Mll5^tm1Apa^* homozygous mating pairs produced offspring, or evidence of pregnancies. To determine whether one or both sexes were affected, we first analyzed reciprocal matings of wild type males with homozygous females and wild type females with homozygous males over a 4-month period. *Mll5^tm1Apa^* female mice are fertile but exhibit a possible rearing defect (Data S1 and Table S1). In contrast, homozygous *Mll5^tm1Apa^* males caged with wild type females did not produce live offspring, nor evidence of pregnancies, showing that homozygous *Mll5^tm1Apa^* males are infertile, although secondary sexual characteristic (Data S1), hormone levels (Table S3) and mating behaviour appeared normal. Table 1 shows that no pregnancies were observed from 11 individual homozygous *Mll5^tm1Apa^* male mice even though they had copulated with the female, as evidenced by the presence of a vaginal plug. The rate of pregnancies was significantly lower than that for heterozygous *Mll5^tm1Apa^* male mice (Multinomial exact test, p = 3.2×10^−7^). Heterozygous littermates were able to plug and impregnate female mice with a frequency indistinguishable from wild type (Table 1, Multinomial exact test, p = 0.14).

**Table 1:** *Mll5 -/-* male mice are infertile.

Breeding pairs			
Male	Female	# pairs	# pregnancies a
*Mll5 -/-*	*Mll5 +/+*	11	0
*l5 +/-*	*Mll5 +/+, +/-*	12	23

Breeding pairs			
Male	Female	# pairs	# pregnancies a
*Mll5 -/-*	*Mll5 +/+, +/-*	11	0
*Mll5 +/+*	*Mll5 -/-*	14	17
*Mll5 +/+*	*Mll5 +/+*	10	22

a Pairs comprising one male and one female were set up for 16 weeks. Only cages in which an initial plug was recorded were included in the experiment. (Top) *Mll5 +/-* male mice are able to produce offspring at a significantly higher rate than *Mll5 -/-* male mice (Binomial exact test, p = 3.2×10^−7^). (Bottom) Breeding rates for *Mll5 +/-* and similar to that of wildtypes (Multinomial exact test, p = 0.14).

### Mll5 appears to be expressed in testes – the site of male gametogenesis

**Figure 1:**
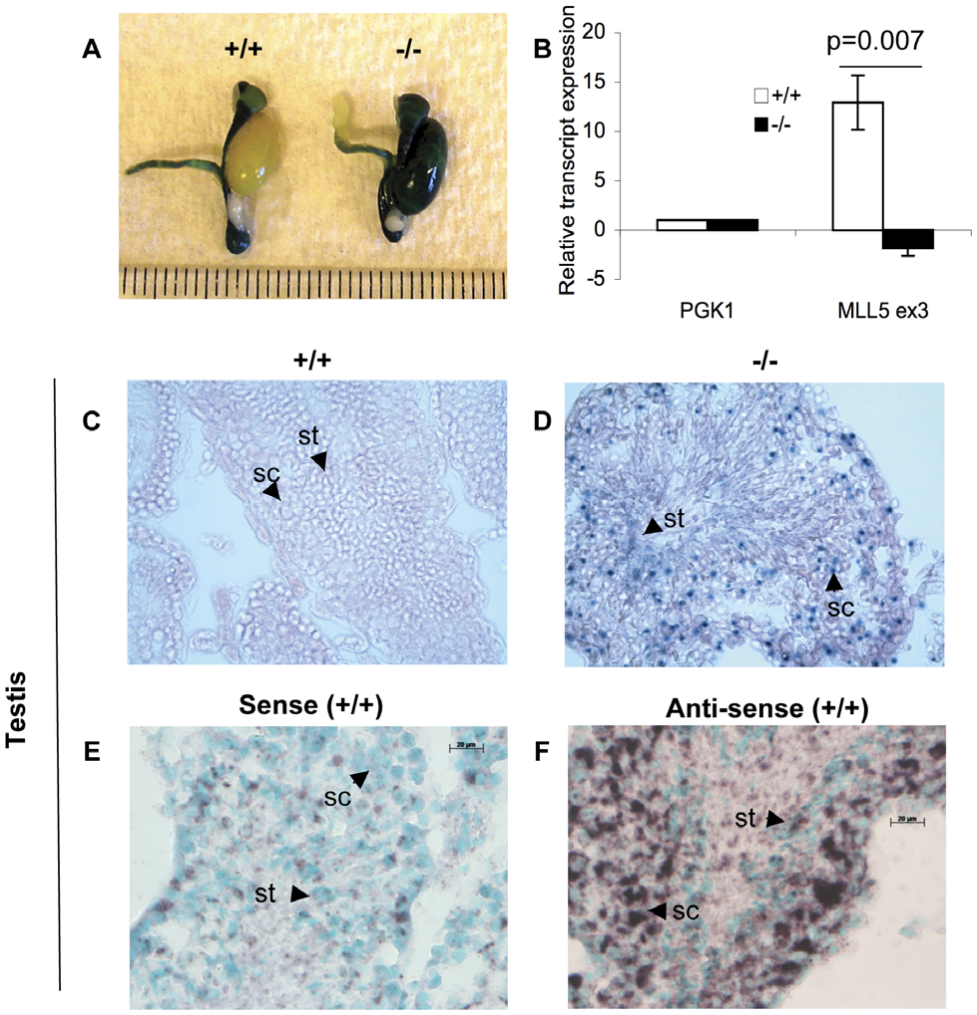
Mll5 is expressed in the developing germ cells of the testes. (A) Whole mount β-galactosidase (β-Gal) staining of wild type (+/+) and homozygous Mll5 ^tm1Apa^ (-/-) testes. Testes from euthanized mice were dissected, fixed and stained with X-Gal. Image was captured with a Nikon S8 and each gradation represents 1mm. (B) Relative expression as quantified by RT-qPCR using SYBR green dye of the wild type *Mll5* transcript as compared to *pgk1* in RNA from whole testes of wild type (+/+) and homozygous *Mll5 ^tm1Apa^* (-/-) mice. The primers that amplify *Mll5* mRNA bind within the region of exon 3 that is deleted (and replaced by a β-Gal cassette) in *Mll5 ^tm1Apa^* [6]. Testes sections of +/+ (C) and -/- (D) mice stained with X-Gal. (E-F) RNA in situ hybridization of +/+ testes sections using *Mll5* RNA probe within exon 3. The scale bar represents 20 µm.

As current antibodies against Mll5 do not work well on tissue sections (data not shown), we used RT-qPCR, β-Galactosidase (β-Gal) staining and RNA in-situ hybridization to assay and visualize *Mll5* expression. The *Mll5^tm1Apa^* allele contains the β-Galactosidase (β-Gal) reporter within exon 3 downstream of an internal ribosome entry site (IRES), allowing for expression of the β-Gal enzyme when the *Mll5^tm1Apa^* transcript is synthesized from the endogenous *Mll5* promoter. Whole-mount β-Gal staining confirmed the presence of the knock out allele (Figure 1A) and RT-qPCR demonstrated the loss of wild type allele expression (Figure 1B) in the testes of Mll5^tm1Apa^ homozygous mice. Expression of β-Gal by the *Mll5^tm1Apa^* allele in knock out mice and *Mll5* mRNA in wild type mice appear to be mainly in developing spermatocytes (sc) and early spermatids (st) (Figure 1D and 1F, respectively). These data show the expression of *Mll5* in developing germ cells.

### Early gametogenesis appears to be intact in homozygous *Mll5 ^tm1Apa^ male mice*

Given the infertility phenotype of homozygous *Mll5^tm1Apa^* male mice and the expression of *Mll5* in the developing germ cells of wild type mice, it was conceivable that loss of *Mll5* generated by the *Mll5^tm1Apa^* allele might impair spermatogenesis. However, we found no gross morphological differences (Data S1 and Figure S1) and mature spermatozoa could be identified in the testes and epididymides, respectively of both genotypes. Additionally, Sertoli cells and other supporting cells appeared normal and showed no obvious morphologic differences (Figures S1), neither was apoptosis significantly elevated in the testes of homozygous *Mll5^tm1/Apa^* male mice (Data S1 and Table S4). Furthermore, there were no statistically significant differences in the counts of mature sperm from the distal epididymides and vasa deferentia of wild type and homozygous *Mll5^tm1/Apa^* male mice (Table 2), although increased variability in sperm counts from *Mll5^tm1apa^* males was noted.

**Table 2:** Characteristics of sperm from Mll5 +/+ and -/- mice.

	Sperm count^a^	Non-motile Sperm^a^	Abnormal head morphology^b^
	(*p* = 0.32)	(*p* = 0.62)	(*p* = 3×10^−25^)
Genotype	Mean No.	Mean	Mean
Mll5 +/+	1.69 ± 0.25×10^7^	29.50 ± 4.95%	11.70% (24/205)
Mll5 -/-	1.45 ± 0.65×10^7^	47.67 ± 25.97%	58.20% (173/298)

a Sperm from the both epididymides and vasa deferentia of freshly sacrificed male mice (age 4-6 months) were counted on a haemocytometer to determine the concentrations per mouse, following capacitation (for motility studies) or fixation (for sperm counts).

b Sperm head morphology was assessed by light microscopy.

### Homozygous *Mll5^tm1Apa^* male mice show abnormal sperm morphology

**Figure 2:**
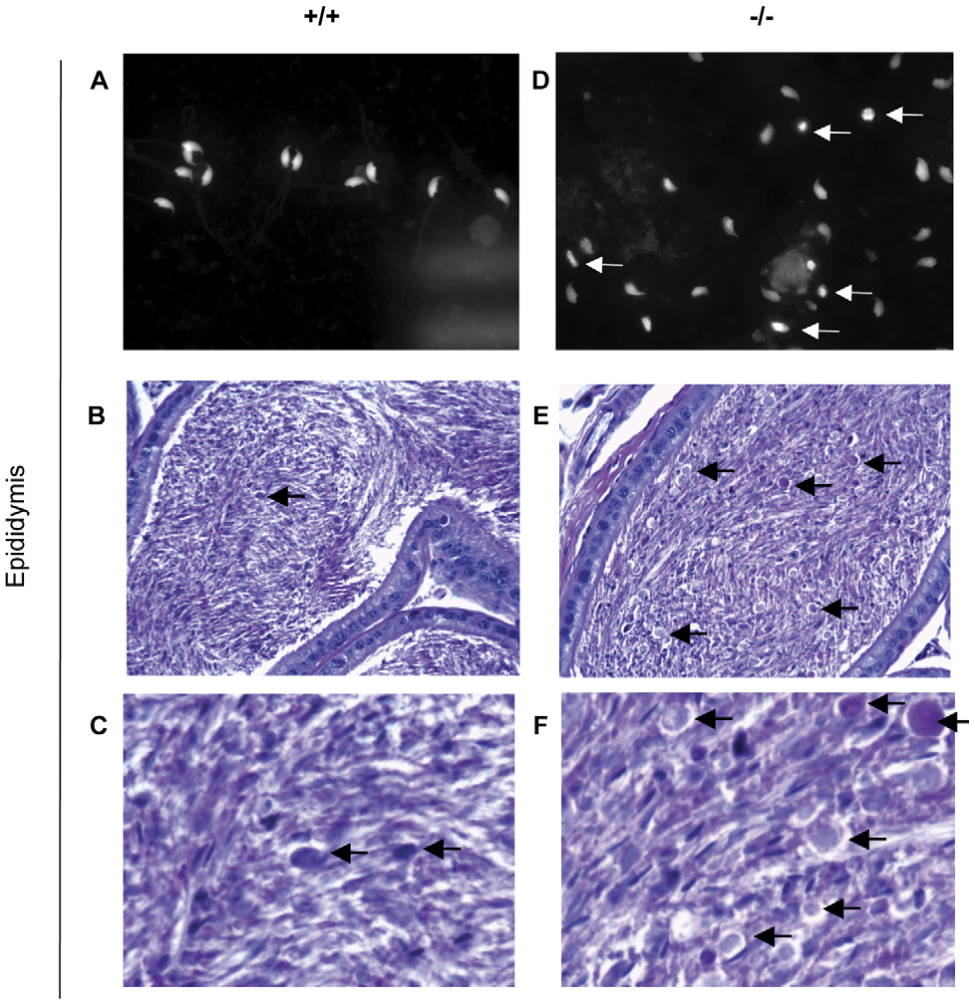
Abnormal spermatozoa in epididymides of homozygous *Mll5^tm1Apa^* mice. Comparison of representative spermatozoa and sections of the epididymides from wild-type (A-C) and *Mll5* -/- (E-F) mice. (A, D) Fluorescence microscopy of spermatozoa from the cauda epididymis and vas deferens with DNA stain (propidium iodide). Abnormally shaped sperm nuclei are arrowed in D. PAS staining shows an excess of cytoplasmic droplets (arrowed) in the cauda epididymides of the mutant males. Original magnification×100 (A–E) while (C, F) are digitally magnified from (B, E) respectively.

The presence of sperm but absence of gross morphological defects in the testis suggested that defects in spermatogenesis might lie in terminal maturation stages and / or capacitation events. We examined periodic acid Schiff (PAS) stained sections of testes and epididymides by light microscopy and observed significant differences between wild type and homozygous *Mll5^tm1Apa^* mice. The corpus epididymides of homozygous *Mll5^tm1Apa^* mice contained a marked increase in quantities of indeterminate, weakly PAS-positive globular material compared with wild types (Figure 2B-C, E-F). In addition, a significantly higher proportion (173/298 = 58%) of spermatozoa collected from the epididymis and vas deferens of homozygous *Mll5^tm1Apa^* mice showed abnormal head morphology as compared to 11.7% (24/205) from wild type mice (Pearson χ^2^_(1)_ = 107, p = 3×10^−25^, Table 2 & Figure 2A, D). These differences suggested that terminal sperm maturation might be affected.

### Homozygous *Mll5^tm1Apa^* male mice show multiple defects in terminal sperm maturation

**Figure 3:**
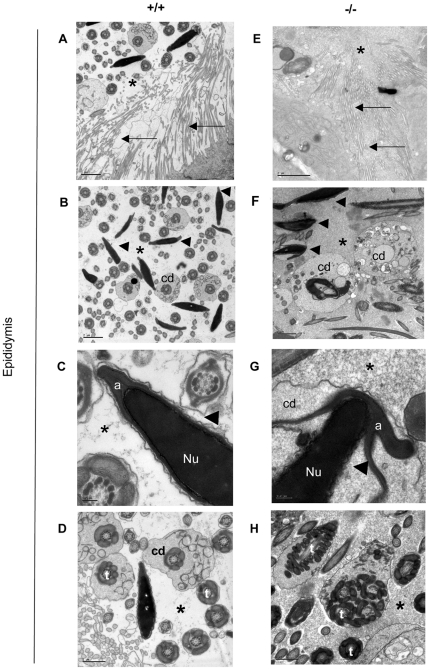
Spermatozoa show multiple subtle defects in homozygous *Mll5^tm1Apa^* mice. Electron micrographs showing spermatozoa in the epididymides of wild type (A–D) and *Mll5 ^tm1Apa^* homozygous males (E–H). In all sections, the lumen is marked by an asterisk (*). The pseudocilia in the caput epididymis are indicated by arrows (A, E). Arrowheads identify the head regions of spermatozoa for comparison (B–C, F–G), while ‘a’ labels the acrosome cap and ‘Nu’ labels the nucleus containing condensed chromatin. Sections of the tail assembly are shown and ‘cd’ marks the cytoplasmic droplet of developing spermatids. The scale bars represent 2 µm (A, B, E, F), 0.2 µm (C, G) and 1 µm (D, H), respectively.

**Figure 4:**
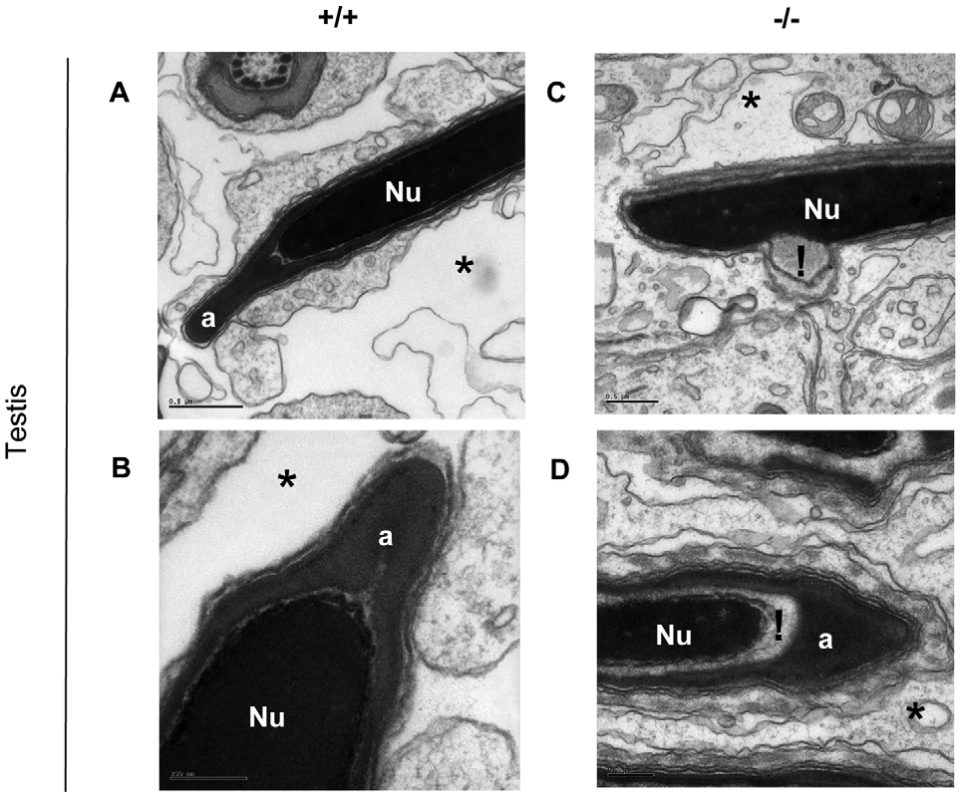
Terminal spermatogenesis is impaired in homozygous *Mll5^tm1Apa^* mice. Morphology of spermatozoa in the seminiferous tubules of testes from wild type (A–B) and *Mll5 ^tm1Apa^* homozygous males (C–D). In all sections, the lumen is marked by an asterisk (*). Electron micrographs showing the head regions of spermatozoa for comparison, with the letters ‘a’ labelling the acrosome cap and ‘Nu’, the nucleus containing condensed chromatin, respectively. Regions of differences in the mutant from the wild type are indicated by ‘!’. The scale bars represent 0.5 µm (A, C) and 0.2 µm (B, D), respectively.

Terminal maturation is a critical step in the production of functional sperm. An electron microscopy survey of spermatozoa present in sixty-three fields from wild-type epididymides and 123 fields from mutant epididymides revealed multiple packaging defects in maturing sperm assemblies from homozygous *Mll5^tm1Apa^* mice that were not seen in the wild type testes. When surveyed at similar locations of the caput epididymis (as identified by the presence of pseudocilia in Figure 3A, E), homozygous mutant sperm heads show greater variability in shape (n = 43 out of 53 sperm from -/-) than wild types (n = 0 out of 23 wild type sperm) (arrowheads in Figure 3B, F). This is accompanied by frequent detachment of the forming acrosome (n = 22/53, labelled ‘a’ in Figure 3C, G) from the nucleus containing the compacted chromatin (labelled ‘Nu’ in Figure 3). Abnormal membranous components and an apparent excess of cytoplasm were seen around some acrosomes of sperm head pieces (n = 20/52, Figure 3F). During the normal terminal packaging of sperm, residual cytoplasm is shed into the cytoplasmic droplet (labelled ‘cd’ in Figure 3) at the tail assemblies of sperm from wild type animals (n = 2 fields out of 6 field containing tail assemblies contained such cases), but excess cytoplasm appeared to remain at the acrosomal regions in sperm of homozygous *Mll5^tm1Apa^* mice (n = 20/53, Figure 3F, G). Furthermore, abundant cytoplasmic droplets in the epididymides of homozygous *Mll5^tm1Apa^* mice were observed (n = 8 fields out of 16 fields containing tail assemblies), in some cases with several tail assemblies pooled into one cytoplasmic droplet (n = 2 out of 8 cases, Figure 3H). We also surveyed terminal maturation in 73 fields from wild type and 23 fields from mutant seminiferous tubules. Evidence of chromatin packaging defects in the nucleus (n = 10/23, Figure 4C), with separation of the acrosomal cap from the nucleus containing condensed chromatin were only observed in homozygous *Mll5^tm1Apa^* mice (n = 8/23, Figure 4D); these anomalies were not observed in wild type mice (n = 73), examined at the same time using the same sampling methodology (Figure 4A, B). Taken together, these observations show that a defect exists in the terminal maturation of spermatozoa in *Mll5^tm1Apa^* homozygous mice.

*In vitro* capacitated sperm isolated from the vasa deferentia of homozygous *Mll5^tm1Apa^* males exhibited lower proportions of sperm not exhibiting motion than wild type males, but failed to reach statistical significance due to the large variations (*Mll5*+/+: 29.5 ± 5.0%, *Mll5*-/-: 47.7 ± 26.0%, ANCOVA F_(1,2)_ = 0.33, p = 0.62; Table 2). Significantly, the movement pattern of sperm that did exhibit motion from mutant mice was significantly slower and showed mostly side-to-side motion, rather than forward swimming, as shown in the attached time-lapse movie (Video S1). Taken together, these data show that Mll5 is required for proper male gametogenesis at the level of terminal maturation of sperm in the testes and/or epididymides.

### The sperm maturation defects exhibited by homozygous *Mll5^tm1Apa^* male mice cannot be rescued by *in vitro* fertilization

**Figure 5:**
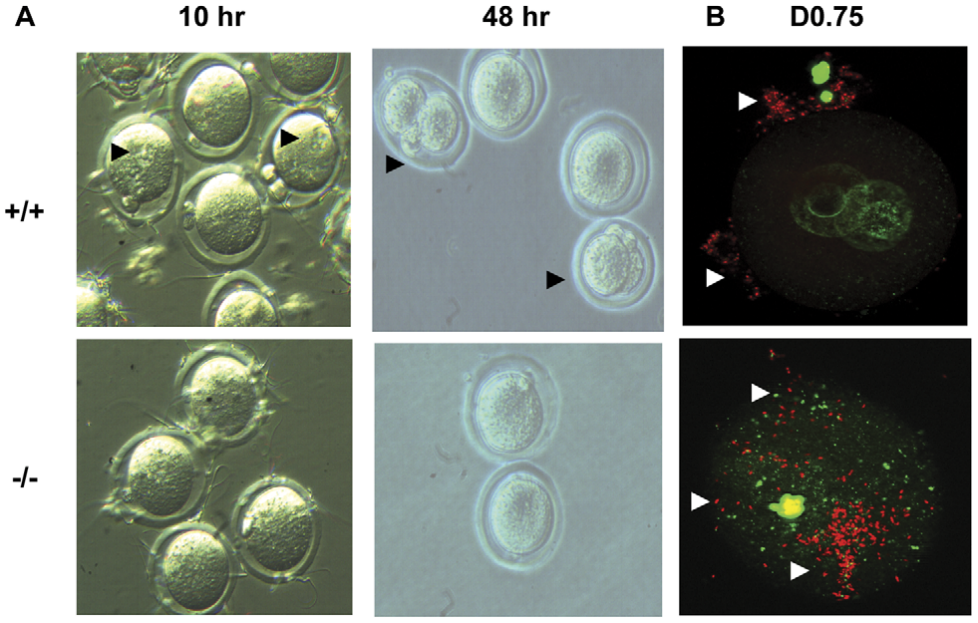
Impaired spermatozoa of homozygous *Mll5^tm1Apa^* mice cannot be rescued by in vitro fertilization. (A) *In vitro* fertilization experiments were performed as described. Arrows indicate male pronuclei (in 10 hr panel) and fertilized embryos (in 48 hour panel) as seen under phase contrast microscopy. The data and percentages are given in Table 3. (B) Embryos flushed from the oviducts of naturally mated females with wild type and -/- males respectively at day 0.75 and were stained with PI (red; DNA) and anti-methyl-Cytosine (green; methylated DNA) and imaged by fluorescence microscopy. Sperm (white arrowheads) can be seen binding to the embryos in both cases, whereas fertilization indicated by the methylation pattern of the two pronuclei is only evident in wild type mated mice.

In spite of the motility defects described above, sperm from homozygous males were found on eggs flushed from oviducts after natural matings (Figure 5B, bottom panel), even though they failed to fertilize the egg; a fertilized egg can be identified by the DNA methylation changes in the two pronuclei as seen in the case of the wild type (Figure 5B, top panel). This suggested that the lack of motility could not fully explain the fertility defect. Moreover, in the homozygous *Mll5^tm1Apa^* mice, 41.8% showed apparently normal morphology under high-powered light microscopy. This raised the question of whether morphologically normal sperm were nevertheless impaired for sperm motility capacitation or the ability to penetrate the zona pellucida and fuse with the egg membrane. We therefore decided to test whether the infertility of homozygous *Mll5^tm1Apa^* mice could be rescued by sperm capacitated using an *in vitro* fertilization procedure. The sperm heads could be observed binding to the oocyte *in vitro* (Figure 5A) as was the case *in vivo* after natural matings (Figure 5B). However, sperm from homozygous males showed a significant (Pearson χ^2^_(2)_ = 67, p = 3x10^−15^) and severe impairment of their ability to fertilize eggs from superovulated wild type females when identical numbers of sperm were incubated with wild type eggs (Table 3). Only three putative fertilization events (3/194, 1.5%) were recorded with homozygous mutant sperm, compared with 28.9% (50/173 for WT 129SvEv) and 34.1% (43/126 for WT ICR) oocytes with sperm from wild type males. Hence, *in vitro* capacitated spermatozoa from homozygous *Mll5^tm1Apa^* mice are impaired in zona pellucida penetration and *in vitro* fertilization. Taken together, the data show evidence for multiple levels of defects in the sperm function of homozygous *Mll5^tm1Apa^* males, including abnormal terminal maturation/packaging, abnormal motility, and inability to penetrate the zona pellucida and fertilize the egg.

**Table 3:** *In vitro* fertilization data.

Genotype & strain of sperm incubated with WT ICR eggs^a^		Total ICR eggs set up	Fertilized (2-cells) embryos (after 1 day)	
Sperm	Strain	Number	Number	% of total^b^
Mll5 -/-	129	194	3	1.5
Mll5 +/+	129	173	50	28.9
Mll5 +/+	ICR	126	43	34.1

a 2.5×10^5^ sperm from each strain and genotype indicated were incubated with wild-type ICR eggs and the fertilization rate calculated by the number of fertilized (2-cell) embryos after 1 day.

b The percentage of fertilized embryos is expressed as the number of 2-cell over the total number of eggs set up in each experiment. Data shown are aggregated from three independent experiments.

### Transcriptional differences between homozygous *Mll5^tm1Apa^* and wild type testes

Given the fact that Mll5 is expressed in the developing germ cells in testes of wild type mice and that spermatogenesis appears to be impaired in homozygous *Mll5^tm1Apa^* males, we sought to define the underlying gene expression differences by assaying for deregulated transcripts in the testes of homozygous *Mll5^tm1apa^* mice. We isolated total RNA from three wild type and three age-matched homozygous *Mll5^tm1apa^* testes and compared the transcriptomes by hybridization to Affymetrix GeneChip Mouse Exon 1.0 Arrays. Three biological replicates for each genotype were compared. We used the exon and transcript (gene) level probeset summarization (Methods) which utilize signals from all exons in a locus to determine transcript level expression. The ratio of expression between wild type and mutant transcripts is summarized in Figure S2. We noted fewer than 961 transcripts showing statistically significant expression differences and short listed genes according the criteria listed in Figure S2 (microarray data from this study may be downloaded at the GEO database with accession number GSE19648). The short listing of transcripts for validation by RT-qPCR came from a longer list of outliers with greater than 1.5 fold change between genotypes and P level of significance<0.05 from both the exon and gene level analyses (Figure S3). We decided to validate by RT-qPCR 27 transcripts (Figure S4) on an additional cohort of three wild type and three homozygous *Mll5^tm1Apa^* testes using Eef1a1 as control probe (Methods) for RNA loading. It has been shown [19]; [20] by global microarray analysis that some commonly used “loading controls” can often be affected by the genotype/treatment and that optimal selection of loading controls can be achieved by from microarray comparisons. Eef1a1 was determined to be the optimal reference probe based on whole microarray analysis of variance with genotype.

**Figure 6:**
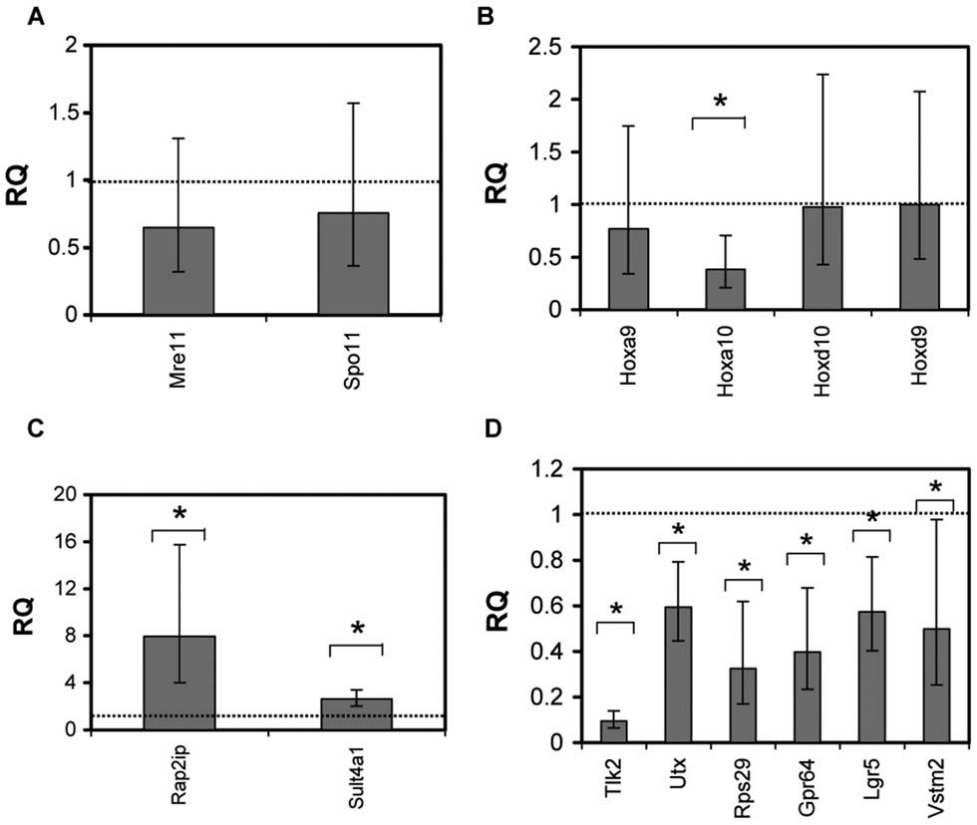
Transcriptional differences in testes of wild type and homozygous *Mll5^tm1Apa^* mice. cDNA from testes of three individual age-matched mice of each genotype were used as input for the Q-PCR. The charts show fold change of *Mll5 -/-* vs wild type (where wild type = 1.0, dotted line) expression levels of the various mRNA as labelled, relative to *Eef1a1*, of genes (A) involved in meiosis, and (B) representative Hox genes and representative genes significantly up regulated (C) and down regulated (D) in -/- testes. Error bars show the 95% upper and 95% lower confidence levels respectively and significant values (p<0.05) and indicated by the asterisks (*).

The short list of transcripts validated included targets from the microarray list as well as other candidate genes such as Hox genes, genes encoding histone modifiers and chromatin proteins as well as genes shown to be misregulated in *Δset3* yeast mutants (Figure S4). We also assayed the expression of transcripts encoding mammalian Spo11 and Mre11 which is involved in mammalian and yeast meiosis [21]–[22]. *SPO11* is prematurely up regulated in SET3-deficient yeast, but neither *Spo11* nor *Mre11* showed statistically significant differences in expression between wild type and homozygous *Mll5^tm1Apa^* mice (Figure 6A), consistent with the previous observation of a predominantly post-meiotic gametogenic defect in homozygous *Mll5^tm1Apa^* mice.

Mll is responsible for the maintenance of Hox gene expression [23]. Furthermore, deregulation of *HoxA10* has been linked to male sterility [24] and *HoxD9* was found to be unregulated in microarray analyses of testes from homozygous *Mll5^tm1apa^* and wild type mice (Figure S3). Hence we postulated that loss of Mll5 might deregulate *Hox* gene expression. RT-qPCR showed *HoxD9* did not show significant expression differences but *HoxA10* was significantly repressed 2.6 fold in homozygous *Mll5^tm1apa^* testes compared with wild type testes (P = 0.0034) (Figure 6B). Expression of adjacent *HoxA9* and paralogous *HoxD10* were not significantly altered (Figure 6B). In addition, we found that two genes, *Rap2ip* and *Sult4A1*, were significantly up regulated 2.6 fold (p = 3.0×10^−9^) and 7.9 fold (p = 2.4×10^−7^) (Figure 6C and Figure S4), respectively in homozygous *Mll5^tm1apa^* testes as compared with testes of the wild type mice. Several transmembrane or putative transmembrane proteins, Gpr64 and Vstm2, which were differentially expressed in microarray analyses (Figure S3), also validated in independent RT-qPCR studies on an additional cohort of mice (Figure S4). These genes had 2.0–2.6 fold lower expression (all p<0.05) in homozygous *Mll5^tm1apa^* testes compared with wild type testes (Figure S4 and Figure 6D). In addition, the gene encoding a testes-expressed kinase [25] involved in meiosis, *Tlk2* was also highly significantly repressed 10.5 fold in homozygous *Mll5^tm1apa^* testes compared with wild type testes (p = 1.9×10^−13^), while transcript *Utx*, encoding a H3K27-specific histone demethylase that associates with Mll3/4 complexes at *Hox* genes [26]; [27] was 1.7 fold lower (p = 0.00082) when *Mll5* was lost (Figure 6D).

## Discussion

In this report, we characterise the phenotype of a loss of function *Mll5* mouse in functional and molecular detail. In addition to the obvious sequences similarities of the SET domain of Mll5 to SET3/4 at critical residues (Figure S5), the reported fertility phenotype herein further suggests that Mll5 may be functionally homologous to yeast SET3 [16]. While both Mll5 and SET3 appear to be important for gamete formation, there are obvious differences; loss of SET3C in yeast results in meiotic failure leading to sporulation defect [18], while functional inactivation of Mll5 in mice leads to post-meiotic spermatogenic defect which results in male sterility.

We also show by RT-qPCR and in-situ RNA hybridization that the *Mll5* is expressed in the testes, particularly in the developing germ cells of wild type mice (Figure 1). Furthermore, the expression pattern of β-Gal by the mutant allele (Figure 1D) very closely reports wild type *Mll5* RNA expression in germ cells (Figure 1F). In addition, our results corroborate and extend previously published findings that show similar *Mll5* expression pattern by northern [28], western blot [7] and microarray data showing expression of *Mll5* primarily in the germ cells (spermatogonia, developing spermatocytes and early spermatids) as well as somatic (Sertoli) cells of mice [29] and rats [30]. Importantly, independently generated *Mll5* knockout mice also show the late stage spermatogenic impairment [7], demonstrating that the presence of foreign elements (in our model) does not alter the phenotype. What the other studies do not address but we show here, is the basis for the sperm defect.

Our breeding experiments show with high statistical confidence that *homozygous Mll5^tm1Apa^* male mice are infertile (Table 1). This is not due to a spermatogenic failure since all stages of spermatogenesis were observed (Figure S1), apoptosis was not significantly increased in *Mll5* -/- testes (Table S4), nor were sperm counts (Table 2) or testes weight (Table S2) significantly reduced in *Mll5* -/- mice. We then go on to show that late stage spermatogenesis or spermiogenesis is impaired in *Mll5* -/- mice. The increased proportion of PAS-stained bodies (Figure 2) and sperm with abnormal head morphology (Table 2, Figure 3) and motility (Video S1) in the testes and epididymides of *Mll5* -/- mice are consistent with this conclusion. Furthermore, genes encoding proteins involved in meiosis, *Spo11* and *Mre11*, were not significantly altered in the testes of homozygous *Mll5^tm1Apa^* mice (as they are deregulated in gametogenesis in yeasts), although detection of premature or delayed expression could be overlooked in our expression analyses of whole testes. Corroborating our conclusion, post-meiotic spermatogenesis has also been observed in an independently generated knock out of *Mll5* [7]. Thus, from the data in homozygous *Mll5^tm1Apa^* mice, it appears that the loss of *Mll5* from developing germ cells results in defects predominantly in the post-meiotic stages of male gametogenesis, although subtle meiotic defects cannot be completely ruled out.

This report also documents for the first time in fine resolution of electron microscopy the structural detail what those defects are. Sperm from the testes and epididymides of Mll5 -/- mice exhibit various defects in spermatozoan maturation such as variations in sperm head morphology, sperm nuclear chromatin compaction and attachment of the nuclear membrane to the acrosome (capping) (Figure 3 & 4). Furthermore, we provide functional evidence that sperm from *Mll5* -/- binds to but is unable to fertilize oocytes in *in vitro* capacitation and fertilization experiments (Figure 5 and Table 3). Such defects are relevant and are consistent with the phenotype.

Thus we conclude that Mll5 is important for normal spermatogenesis as functional inactivation of *Mll5* by constitutive knockout in a mouse exhibits male infertility due to defects in late stage spermatogenesis or spermiogenesis resulting in spermatozoa, which are unable to fertilize oocytes *in vitro* as well as *in vivo*.

Three groups independently targeted *Mll5* in mice and concordantly found that functional inactivation of *Mll5* results in a hematopoietic stem cell defect [6] [17] [7]. In order to discover the targets of Mll5 which would help to elucidate the biochemical and molecular mechanism of how Mll5 works *in vivo*, microarray was performed on flow cytometry-sorted murine bone marrow cells to purify hematopoietic stem cells (HSCs) since that was the population of cells which was found to be defective in the *Mll5* -/- mice [17]. Their microarray did not reveal many differences in the HSCs of wildtype and knockout Mll5 mice apart from *Hoxb2* and *Hoxb5* [31]. In this report, we have shown a clear phenotype in the testes of *Mll5* -/- mice and hence assayed differential expression of genes from the testes of wildtype and Mll5 -/- mice using microarray. Unlike differential expression in hematopoietic stem cells, we were able to find over 900 differentially expressed transcripts between wildtype and Mll5 -/- testes. Several genes were found to be differentially expressed in whole mouse testes from wildtype and Mll5 -/- mice by RT-qPCR analyses on independent batches of mice (Figure 6).

The targets identified in our study may provide some possible avenues for future studies on the mechanism of Mll5, though it remains to be determined if these are direct targets of Mll5. Loss of Mll5 appeared to correlate with the repression of *HoxA10* but not *HoxA9*, *HoxD9* or *HoxD10* (Figure 6B). *HoxA10* knockout mice manifest spermatogenic defects [32]–[33], although obvious cryptorchidism was not observed in homozygous *Mll5^tm1Apa^* mice. Another significantly repressed gene, Gpr64 (Figure 6D) also plays an important role in regulating the osmotic environment for spermatozoa maturation in the epididymis and its loss leads to male sterility reminiscent of homozygous *Mll5^tm1Apa^* [34]. *Tlk2,* which was down regulated in *Mll5* -/- testes, encodes a serine/threonine kinase which shows significant sequence homology to the Tousled kinase in Arabidopsis (hence its name Tousled-like kinase) and is associated with differentiation of the reproductive organs of plants [25]. *Tlk2* mRNA is expressed at high levels in testes, leading to speculation about a role in gametogenesis in mammals, as in plants [35]. Rap2ip (synonyms RPIP8, Rundc3a) was up regulated in both the microarray analyses (Figure S4) and the RT-qPCR validation where it was up regulated 7.9 fold in testes *mll5^tm1Apa^* mice, implicating the Ras-signalling pathway [36] in male fertility [37]. *Sult4a1*, which was up regulated over 2 fold in the testes of *mll5^tm1Apa^* mice in the microarray (Figure S3) and RT-qPCR analyses (Figure 6C), encodes a cytosolic sulfotransferase enzyme which is believed to modulate the function of endogenous catecholamines and steroid hormones in the brain [38] however an endogenous substrate for Sult4a1 has not been identified [39]; [40] nor has its role in spermatogenesis been determined.

In this report, we have characterised the role of murine Mll5 in normal gametogenesis. Homozygous *Mll5^tm1apa^* mice show impaired late-stage spermatogenesis or spermiogenesis. We have also shown that of the transcriptional differences, several targets are known to be involved in spermatogenesis. These and other targets may explain the phenotype. The mechanisms of the deregulated transcript expression are unknown at present, although it is possible that histone modifications might be involved, given the reported function of Mll5 and the other Mll family members. ChIP grade antibodies have so far proven intractable for Mll5, preventing direct ChIP strategies to analyze the promoters of these genes, however their identification in this report defines them as possible targets for future analysis. The genetic models of Mll5 define it as an essential protein for normal spermatogenesis and haematopoiesis and thus future studies are required to define the complexes in which Mll5 operates and the testes model should aid in those biochemical studies.

## Methods

### Mouse breeding

All mice were bred and maintained as approved by the University of British Columbia Animal Care Committee (A05-0699) or under the authority of a U.K. Home Office Project License (PPL80/1503). The transgenic mice (*Mll5^tm1Apa^*) were maintained as an inbred stock on a 129S6 (129SvEv) genetic background on a high-fat sterile diet. Mating pairs were supplemented with dough diet and sunflower seeds. Genotyping of mice was done by PCR using the primers as described elsewhere [6].

### Experiments on mice

For experimental matings, male mice were singly housed overnight before the female was introduced and monitored for the presence of a plug. Mice were euthanized by raising CO_2_ concentration and blood and/or tissues collected as specified. For the removal of embryos, oviducts or uteri of freshly sacrificed female mice were dissected and then flushed with M2 media (Millipore, MA), incubated with hyaluronidase (Sigma, 10 mg/ml in M2) and washed in M2 or Human Tubal Fluid (HTF) (Millipore, MA).

### Staining and hybridization

Testes from euthanized mice were dissected, fixed in 4% paraformaldehyde and stained overnight at 30°C with X-Gal. RNA in situ hybridization was performed on 10–15 µm sections of frozen testes using a 150-bp *Mll5* RNA probe within exon 3 using the protocol described before [41] (Methods S1).

### Antibody staining

Embryo staining was performed as previously described [42]. Freshly dissected embryos were washed in phosphate buffer solution (PBS) and fixed overnight at 4°C in 4% paraformaldehyde with Polyvinyl alcohol (PVA) (0.1 mg/ml). After fixation, embryos were washed in 0.05% Tween 20 (VWR) and permeabilized in 0.2% Triton X-100 (VWR) for 30 min and after washes in 0.05% Tween 20, incubated for 1 hr at 37°C in 2 N hydrochloric acid. After washing, embryos were incubated with 2% bovine serum albumin (BSA) and methylated DNA was visualized with mouse anti-5-methylcytosine (Calbiochem) (1/500, 1 hr at 37°C) and FITC-conjugated anti-mouse IgG (eBioscience) (1/100, 1 hr at room temperature). DNA was visualized by propidium iodide staining in the mounting medium (Vector Laboratories).

### In vitro fertilization experiments

Female ICR mice were superovulated with an intraperitoneal (i.p.) injection of 1.5U pregnant mare serum (PMS, Sigma), followed 48 hr later by an ip injection of 1.5U human chorionic gonadotropin (hCG, Sigma). Thirteen hours after hCG administration, the superovulated female mice were euthanized and the oocytes were harvested and incubated for 4–6 hour with 2.5×10^5^ sperm (diluted to 1−25×10^6^ / ml in capacitation buffer for 30 min before mixing with oocytes) from epididymides and vasa deferentia of wild type (Mll5 +/+) and homozygous *Mll5^tm1Apa^* (Mll5 -/-). (ICR strain oocytes but not oocytes from 129S6 mice could be reproducibly fertilized *in vitro* by sperm of wild type males from both strains). For assessment of fertilization, the number of two cell embryos with an extruded second polar body, 24 hours after fertilization was recorded.

### Characterization of mouse sperm

Testes from euthanized male (n = 3 per genotype) mice were removed and the epididymides and vasa deferentia dissected into 3 ml of DMEM and incubated at 37°C / 5% CO_2_ for 30 min to allow for capacitation of the sperm. An aliquot was then taken and non-motile sperm was counted using a haemocytometer while another aliquot taken to observe sperm using time-lapse microscopy on a Zeiss Colibri AxioObserver.Z1. A third aliquot was fixed in 4% paraformaldehyde and the total number of sperm counted. The number of non-motile sperm was then expressed as a percentage of the total number of sperm. At least 100 single sperm per mouse were analyzed. For imaging, propidium iodide was used in the mounting media and the sperm heads imaged using a Nikon C1 TE2000E2 confocal microscope with a 63x objective.

### Electron microscopy

Freshly obtained tissues were fixed in 2.5% glutaraldehyde in a 0.1 M Cacodylate buffer at pH 7.3 for 2 hr and washed in the same buffer lacking glutaraldehyde three times. Next they were fixed in 1% osmium tetroxide and potassium ferrocyanide 1% in the same Cacodylate buffer for one hour followed three rinses in distilled water before being dehydrated through a graded series of Acetone to 100% starting at 30%. After two changes in propylene oxide they were infiltrated with epon 812 and then embedded in the same epoxy resin. 60 to 70 nm thin sections were viewed in the FEI Tecnai 12 Transmission Electron Microscope.

### Microarray Analysis and Validation

Total RNA was isolated from three frozen wild type and age-matched homozygous *Mll5^tm1apa^* testes using QIAzol™ lysis reagent (Qiagen, Maryland, USA). RNA was extracted according to the manufacturer's instructions (Methods S1) and probed on GeneChip Mouse Exon Array 1.0 ST chips (Affymetrix, Santa Clara, California) for gene expression analysis (Methods S1). Raw intensity calls were normalized using quantile normalization [43] and probeset summarization (core plus extended) undertaken with gc-rma. Outliers with greater than 1.5 fold change between genotypes and level of significance, p<0.05 were selected for further analysis by quantitative PCR (qPCR) (Figure S3).

For validation, quantitative real-time polymerase chain reaction (RT-qPCR) was set up in 384 well plates on the 7900HT Fast Real-Time PCR system (Applied Biosystems, Foster City, CA) with the respective probes (Roche) and primers (Figure S4 and Methods S1). Relative quantification (RQ) of the mutants compared to wild type was determined as 2^-ΔΔC^_t_ , with ΔΔC_t_ obtained from the parameters of the linear mixed effects model. Tests for genotype effect on gene of interest adjusted for amplification using the endogenous control were carried out using the likelihood ratio test.

### Statistical analysis

Pairwise comparisons were performed using Student's *t* test for continuous variables, and Pearson's chi squared test or Fisher's exact test for categorical variables. Results with a 2-sided *P-*value less than 0.05 were considered significant. For birth rate variables with a Poisson distribution, birth counts conditional on the total births have a multinomial distribution, from which exact p-values were obtained [44]. For the panel of genes assessed via RT-qPCR, the Benjamini-Hochberg method was used to adjust p-values for multiple comparisons. Only gene targets with adjusted p-values<0.05 were declared significantly differentially expressed. Unadjusted p-values are reported to allow their use in possible future meta-analyses. Statistical analyses were performed with Excel (Microsoft Canada, Mississauga, ON) and the R statistical package (R Foundation for Statistical Computing, Vienna, Austria). Microarray analysis was performed using ArrayAssist version 5.0 (Stratagene, California USA). All microarray data is MIAME compliant and the raw data has been deposited in a MIAME compliant database (GEO accession GSE19648).

## Supporting Information

Figure S1 **Gametogenesis is grossly normal in homozygous** ***Mll5^tm1Apa^*** **mice.** Representative sections of haematoxylin and eosin (H & E) stained seminiferous tubules in testis of wildtype (A–B) and *Mll5 -/-* (E–F) mice showing various different cell types. 1 – spermatogonia; 2 – spermatocytes; 3 – round spermatids; 4 – elongating spermatids; 5 – Sertoli cells, 6 – Leydig cells. Representative sections of H & E stained epididymides of wild-type (C–D) and *Mll5 -/-* (G–H) mice showing (1) coiled tubules of the epididymis which are (2) lined by columnar epithelium and (3) contain mature spermatozoa. The original magnification was X400 for all panels except for C and G, where the magnification was X100.
(TIF)

Figure S2 **Representation of Microarray data showing outliers in yellow.** Plot of fold change (FC) of transcripts against their respective p-values of the transcriptomes of homozygous *Mll5 ^tm1Apa^* (KO) to *Mll5* +/+ (WT) testes as determined by microarray. The yellow dots show the outliers and transcripts with FC>1.05 and p<0.05.
(TIF)

Figure S3 **Filtered results from the analysis of the Affymetrix Microarray and status of RT-qPCR validation.** Comparisons between genes identified as differentially expressed by microarray and their location on the mouse genome according to Mus musculus, NCBI build 37, 2007-07 from the Core Gene Level analysis between *Mll5* -/- (KO) and *Mll5* +/+ (WT) testes are shown.
(TIF)

Figure S4 **Validation of short listed genes by RT-qPCR.** This table shows the transcripts, primers and probes used (SYBR = SYBR green used without UPL probes) as well as the relative expression of transcripts in testes of homozygous *Mll5 ^tm1Apa^* [KO] mice vs wild type [WT] mice. Up regulated transcripts are highlighted in red while down regulated transcripts are highlighted in green. P values that are significant are in red text.
(PDF)

Figure S5 **Mll5 appears to have similar residues in the SET domain to yeast SET3/4.** CLUSTAL 2.0.8 multiple sequence alignment of the SET domains of Mll1(KMT2A) (**P552200**), Mll4(Wbp7,KMT2D) (**O08550**), Mll2 (KMT2B) (**Q6PDK2**), Mll3(KMT2C) (**QBR4H**), Mll5(KMT2E) (**Q3UG20**), yeast SET3 (**P36124**) yeast SET4 (**P42948**) and Setd7 (**Q8VHL1**). Conserved structural residues are marked in green and residues important for catalysis in red. Critical residues not conserved are marked in purple.
(TIF)

Table S1 ***Mll5 -/-*** **female mice are fertile.**
(DOC)

Table S2 **Mean testes weights from Mll5 +/+ and -/- mice.**
(DOC)

Table S3 **Mean testosterone levels in Mll5 +/+ and -/- mice.**
(DOC)

Table S4 **Apoptosis in Testes from Mll5 +/+ and -/- mice.**
(DOC)

Data S1 **Supplemental data.**
(DOC)

Methods S1 **Supplemental methods.**
(DOC)

Video S1 ***Mll5*** **-/- female mice are fertile.** Time-lapse video microscopy showing differences in motility of sperm from Mll5 +/+ and Mll5 -/- mice.
(MP4)
