# Supplementary figures and images for: Mll5 Is Required for Normal Spermatogenesis
Source: PLoS One. 2011 Nov 1;6(11):e27127. doi: 10.1371/journal.pone.0027127 (PMC3206077; doi:10.1371/journal.pone.0027127)

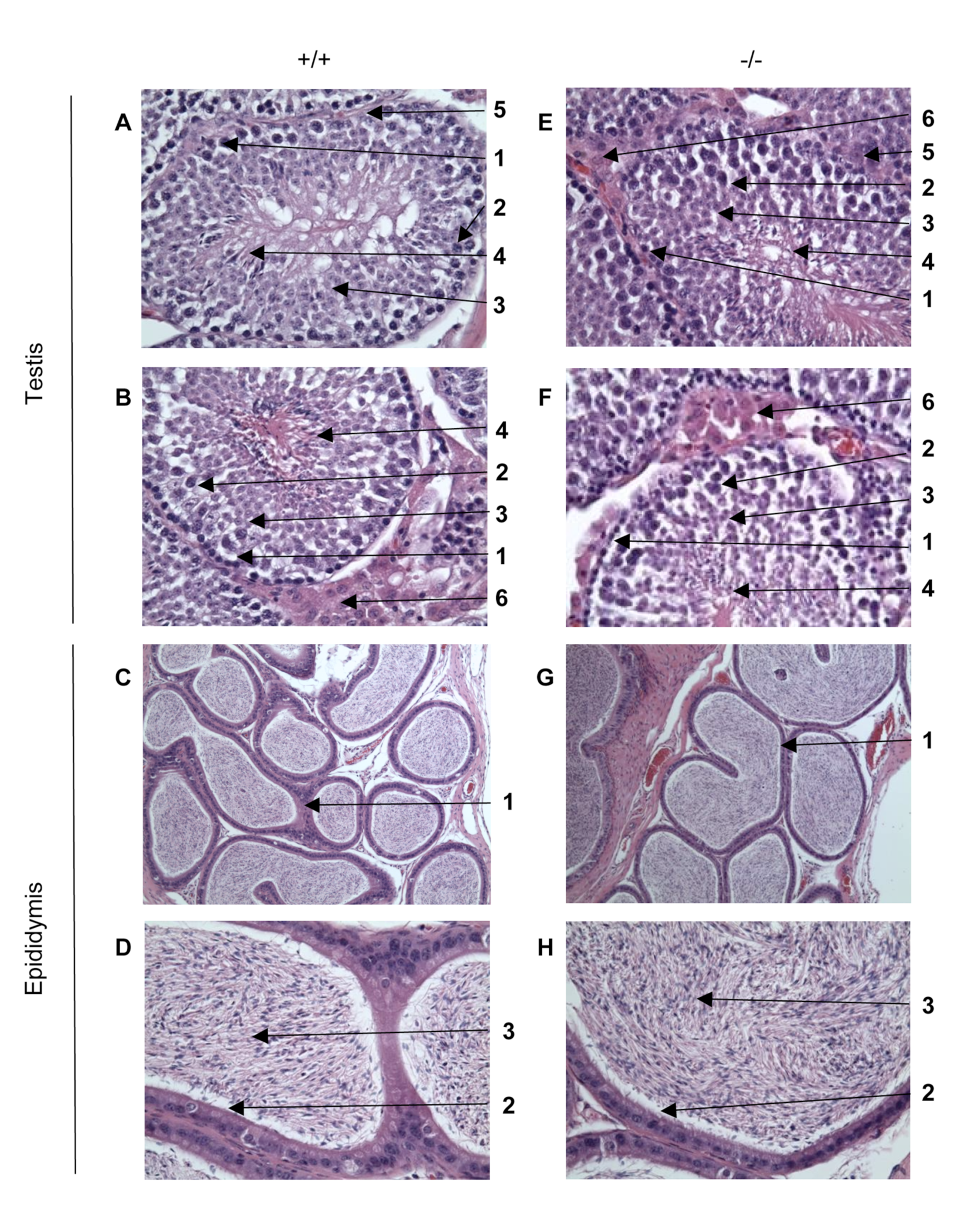

Supplement: Figure S1 — Gametogenesis is grossly normal in homozygous Mll5tm1Apa mice. Representative sections of haematoxylin and eosin (H & E) stained seminiferous tubules in testis of wildtype (A–B) and Mll5 -/- (E–F) mice showing various different cell types. 1 – spermatogonia; 2 – spermatocytes; 3 – round spermatids; 4 – elongating spermatids; 5 – Sertoli cells, 6 – Leydig cells. Representative sections of H & E stained epididymides of wild-type (C–D) and Mll5 -/- (G–H) mice showing (1) coiled tubules of the epididymis which are (2) lined by columnar epithelium and (3) contain mature spermatozoa. The original magnification was X400 for all panels except for C and G, where the magnification was X100. (TIF) [file pone.0027127.s001.tif]

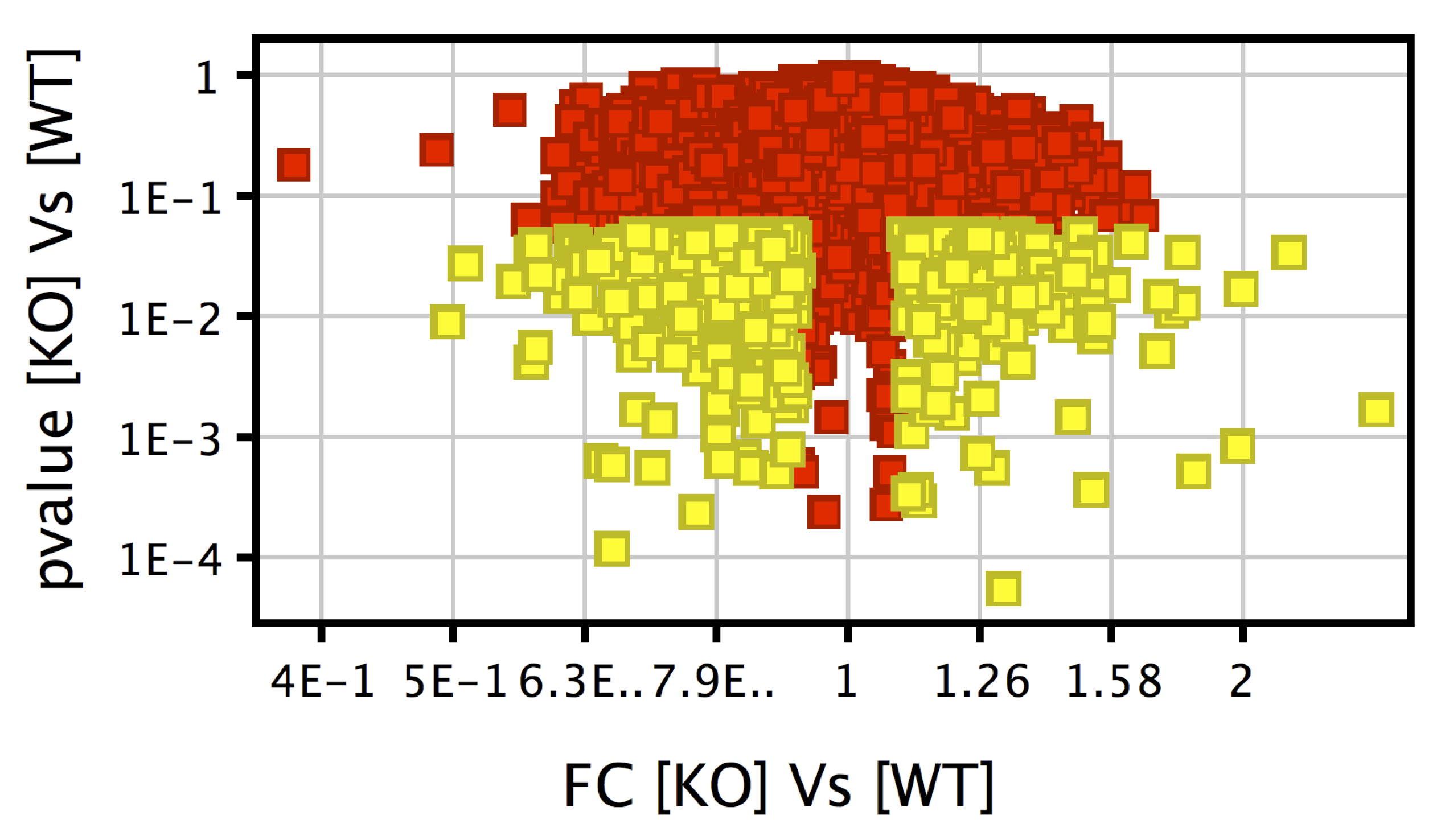

Supplement: Figure S2 — Representation of Microarray data showing outliers in yellow. Plot of fold change (FC) of transcripts against their respective p-values of the transcriptomes of homozygous Mll5 tm1Apa (KO) to Mll5 +/+ (WT) testes as determined by microarray. The yellow dots show the outliers and transcripts with FC>1.05 and p<0.05. (TIF) [file pone.0027127.s002.tif]

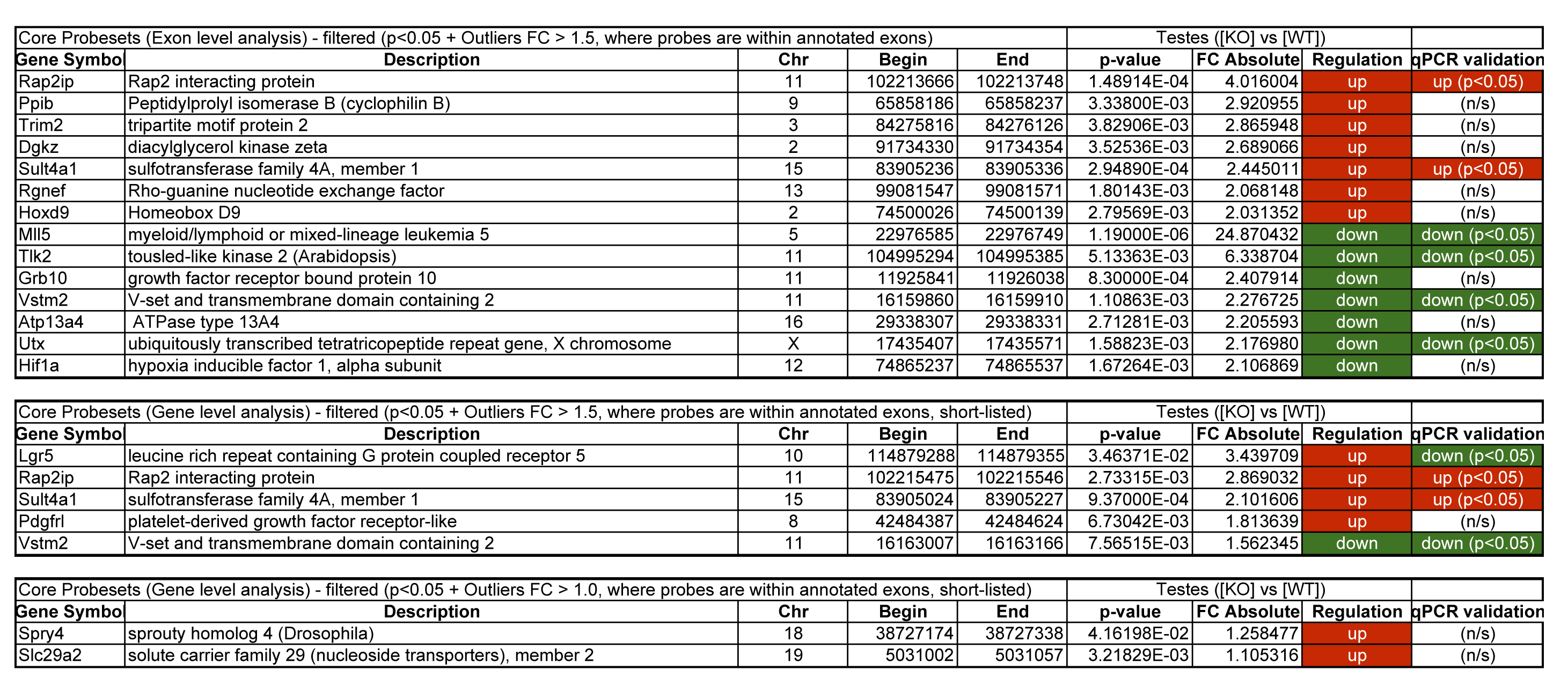

Supplement: Figure S3 — Filtered results from the analysis of the Affymetrix Microarray and status of RT-qPCR validation. Comparisons between genes identified as differentially expressed by microarray and their location on the mouse genome according to Mus musculus, NCBI build 37, 2007-07 from the Core Gene Level analysis between Mll5 -/- (KO) and Mll5 +/+ (WT) testes are shown. (TIF) [file pone.0027127.s003.tif]

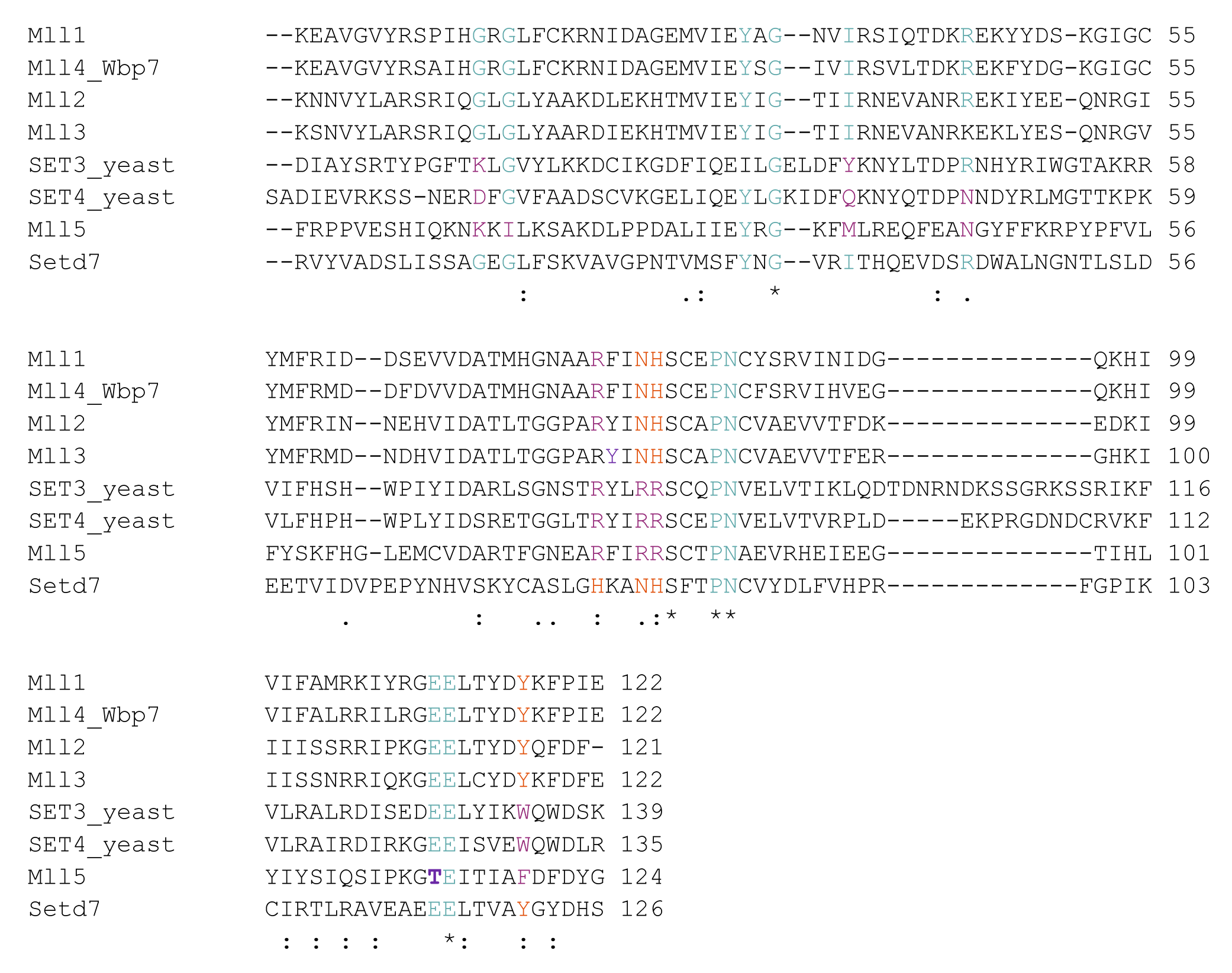

Supplement: Figure S5 — Mll5 appears to have similar residues in the SET domain to yeast SET3/4. CLUSTAL 2.0.8 multiple sequence alignment of the SET domains of Mll1(KMT2A) (P552200), Mll4(Wbp7,KMT2D) (O08550), Mll2 (KMT2B) (Q6PDK2), Mll3(KMT2C) (QBR4H), Mll5(KMT2E) (Q3UG20), yeast SET3 (P36124) yeast SET4 (P42948) and Setd7 (Q8VHL1). Conserved structural residues are marked in green and residues important for catalysis in red. Critical residues not conserved are marked in purple. (TIF) [file pone.0027127.s005.tif]
