# Supplementary material for: Mll5 Is Required for Normal Spermatogenesis
Source: PLoS One. 2011 Nov 1;6(11):e27127. doi: 10.1371/journal.pone.0027127 (PMC3206077; doi:10.1371/journal.pone.0027127)
Supplement: Figure S4 — Validation of short listed genes by RT-qPCR. This table shows the transcripts, primers and probes used (SYBR = SYBR green used without UPL probes) as well as the relative expression of transcripts in testes of homozygous Mll5 tm1Apa [KO] mice vs wild type [WT] mice. Up regulated transcripts are highlighted in red while down regulated transcripts are highlighted in green. P values that are significant are in red text. (PDF) [file pone.0027127.s004.pdf]

	GeneTarget	Primer 1	Primer 2	DetectionUPL probe or SYBR green	KO/WT FCest	Pr(>Chisq)	KO/WT FC 95% LCL	KO/WT FC 95% UCL	KO/WT Abs FCest	KO/WT Abs FC Direction	KO/WT Abs FC 95% LCL	KO/WT Abs FC 95% UCL
1	Tlk2	ctcaggaaacaggcatcagg	tgtctgaacgtgtctgaagga	UPL17	0.094649768	1.91E-13	0.064136	0.139681	10.56527	Down	7.159192	15.59182
2	Utx	cgtgaggtttcatgaagatgg	gagattcgtagcagcgaaca	UPL3	0.59499958	0.00082	0.447026	0.791956	1.680673	Down	1.262697	2.237008
3	Rps29	ctgaaggcaagatgggtca	gcacatgttcagcccgtatt	UPL109	0.324657825	0.00124	0.17012	0.619577	3.080166	Down	1.614003	5.878193
4	Gpr64	tcaagatacagctaatggcacct	gagttttgttgagctctgatcg	UPL109	0.398017564	0.001321	0.233627	0.678082	2.512452	Down	1.474749	4.280332
5	lgr5	tcctcacttgacaggaactgc	gaagagatgagatctttgctcca	UPL80	0.573618406	0.002927	0.403662	0.815133	1.743319	Down	1.226793	2.477322
6	Hoxa10	ccttcagaaaacagtaaagcttcg	aagggcagcgtttcttcc	UPL100	0.383136982	0.003407	0.207128	0.708711	2.610033	Down	1.411013	4.827928
7	vstm2	ccagaggacctggagcaa	cgggtctctgtcgggtaag	UPL75	0.497692367	0.037403	0.253129	0.978543	2.009273	Down	1.021927	3.950555
8	Grb10	cggttgctcctagctcctta	tgaaagcctggagggaaat	UPL83	0.570590514	0.094375	0.287976	1.130557	1.75257	Down	0.88452	3.472509
9	Hif1a	aatggaacggagcaaaagac	tgtggtaatccactctcatcca	UPL3	0.504257427	0.101347	0.214966	1.182866	1.983114	Down	0.845405	4.651905
10	Atp13a4	tctcctgggcaaaagtcaag	gcaccatctccacacatacct	UPL31	0.583127056	0.146588	0.272413	1.248243	1.714892	Down	0.801126	3.670901
11	Trim2	ggaggtcaactatggtctcaaac	gaagttgctgcagctcttaatg	UPL72	0.580593436	0.148717	0.268448	1.255696	1.722376	Down	0.796371	3.725119
12	Ppib	cttcataaccacagtcaagacctc	ctccaccttccgtaccacat	UPL42	0.603538622	0.151013	0.30007	1.213915	1.656895	Down	0.823781	3.332561
13	Mre11	ccggaagctcagtggtga	aatgcgcagcaagcctac	UPL80	0.646353657	0.203495	0.319232	1.308681	1.547141	Down	0.764128	3.132515
14	Rpl4	agcagccgggtagagagg	atgactctccctttttcggagt	UPL55	0.689075276	0.354859	0.299933	1.583102	1.45122	Down	0.631671	3.334077
15	sprx	caacaaggcctggagtgg	gggcacttgattctaggaggt	UPL75	0.750096353	0.443395	0.344974	1.630978	1.333162	Down	0.613129	2.898772
16	Spo11	ggctcctggacgacaactt	cagatctggaacgcccttt	UPL18	0.754411985	0.448996	0.362435	1.570315	1.325536	Down	0.636815	2.759115
17	Hoxa9	tccctgactgactatgcttgtg	gttggcagccgggttatt	UPL25	0.769580065	0.507932	0.338985	1.747138	1.29941	Down	0.572365	2.949983
18	Pgdfr1	ccaaaacaggctccacctat	cgggttcaggtagacaacatc	UPL7	0.800711908	0.542305	0.375851	1.705836	1.248889	Down	0.586223	2.660631
19	Rgnef	ccagcgaagaacagagatca	cccattcttcttcctgaatcc	UPL2	0.866490998	0.738122	0.35571	2.110729	1.15408	Down	0.47377	2.811281
20	dgk2	gcctccctcatgaagacaga	cgctggatcatctggtaatg	UPL80	0.95164034	0.881132	0.477799	1.895398	1.050817	Down	0.527594	2.09293
21	Hoxd10	ctgaggtttccgtgtccagt	ttggagtatcagacttgatttcctc	UPL2	0.976672987	0.952775	0.426507	2.236518	1.023884	Down	0.447124	2.344628
22	Sult4a	ggggctggacatcatcaa	agagggcaggaagcggta	UPL10	2.630203534	3.00E-09	2.027477	3.412108	2.630204	Up	2.027477	3.412108
23	Rap2IP	agctttgtccagaccaccat	tctagcaggttttcacagaga	UPL45	7.948129249	2.36E-07	4.007745	15.76267	7.948129	Up	4.007745	15.76267
24	Slc2a9	ttccaacggctacttggtgt	aggctccgcaggagagtc	UPL72	2.038252098	0.10701	0.827093	5.022982	2.038252	Up	0.827093	5.022982
25	Spry4	tacacagacgtggagcgatg	caccaagggacaggcttcta	UPL17	1.002000959	0.985829	0.797453	1.259015	1.002001	Up	0.797453	1.259015
26	Hoxd9	ctcagcttgcagcgatca	ttcgctgcaggttgttt	UPL63	1.00138552	0.996851	0.482949	2.076355	1.001386	Up	0.482949	2.076355
27	Eef1a1	acacgtagattccggcaagt	aggagccctttcccatctc	UPL31	Endogenous control							
28	Mll5	tcagcacatctgaagatggaa	caggaatatgctgcctgtca	SYBR	0.000417317	0.006623				Down		
29	Pgk1	tacctgctggctggatgg	cacagcctcggcatatttct	SYBR	Endogenous control							
