# Supplementary material for: Mll5 Is Required for Normal Spermatogenesis
Source: PLoS One. 2011 Nov 1;6(11):e27127. doi: 10.1371/journal.pone.0027127 (PMC3206077; doi:10.1371/journal.pone.0027127)
Supplement: Table S1 — Mll5 -/- female mice are fertile. (DOC) [file pone.0027127.s006.doc]

## Table S1. *^Mll5^* ^-/- female mice are fertile^.

| **Breeding pairs** | | |  |  |  |
| --- | --- | --- | --- | --- | --- |
| **Male** | **Female** | **# pairs** | **# pregnancies ^a^** | **# mice born** | **# mice weaned** |
| *Mll5 +/+* | *Mll5 -/-* | 14 | 17 | 76 | 8 ^b^ |
| *Mll5 +/+* | *Mll5 +/+* | 10 | 22 | 106 | 96 |

^a^ Pairs comprising one male and one female were set up for 16 weeks. Only cages in which an initial plug was recorded were included in the experiment.

^b^ Of the 8 mice weaned, four were from the third litter of a *Mll5* -/- mouse and four were fostered to a DR4 mother.
