# Supplementary material for: Mll5 Is Required for Normal Spermatogenesis
Source: PLoS One. 2011 Nov 1;6(11):e27127. doi: 10.1371/journal.pone.0027127 (PMC3206077; doi:10.1371/journal.pone.0027127)
Supplement: Table S2 — Mean testes weights from Mll5 +/+ and -/- mice. (DOC) [file pone.0027127.s007.doc]

## Table S2. Mean testes weights ^a^ from Mll5 +/+ and -/- mice.

| **Genotype** | ***n =*** | **Mean Weight** | **SD** | ***p =*** |
| --- | --- | --- | --- | --- |
| Mll5 -/- | 13 | 0.1741 g | 0.0430 g | 0.96 |
| Mll5 +/+ | 14 | 0.1732 g | 0.0460 g |

^a^ Both testes were harvested from aged matched male mice (age 6-12 months) and weighed. Table shows the mean testis weight per animal.
