# Supplementary material for: Mll5 Is Required for Normal Spermatogenesis
Source: PLoS One. 2011 Nov 1;6(11):e27127. doi: 10.1371/journal.pone.0027127 (PMC3206077; doi:10.1371/journal.pone.0027127)
Supplement: Table S3 — Mean testosterone levels in Mll5 +/+ and -/- mice. (DOC) [file pone.0027127.s008.doc]

## Table S3. Mean testosterone levels ^a^ in Mll5 +/+ and -/- mice.

| **Genotype** | ***n =*** | **(T) Mean** | **SD** | ***p =*** |
| --- | --- | --- | --- | --- |
| Mll5 -/- | 13 | 121.5 | 253.1 | 0.96 |
| Mll5 +/+ | 15 | 153.7 | 296.3 |  |

^a^ Free serum testosterone (T) of wild type and -/- mice. Mice were singly housed for a week before being euthanized by rising CO_2_ concentration. Blood was obtained by cardiac puncture. Serum of Mll5 +/+ and -/- was collected as the supernatant after centrifugation of collected blood. Free testosterone was measured using an ELISA kit (DB52181; IBL, Hamburg, Germany), according to the manufacturer’s instructions. Sensitivity is 0.17pg/ml with 8.9% intrassay variation and 8.8% interassay variation.

## 
