# Supplementary material for: Mll5 Is Required for Normal Spermatogenesis
Source: PLoS One. 2011 Nov 1;6(11):e27127. doi: 10.1371/journal.pone.0027127 (PMC3206077; doi:10.1371/journal.pone.0027127)
Supplement: Table S4 — Apoptosis in Testes from Mll5 +/+ and -/- mice. (DOC) [file pone.0027127.s009.doc]

## Table S4. Apoptosis^a^ in Testes from Mll5 +/+ and -/- mice.

| **Genotype** | ***n =*** | **No of +ve nuclei ^b^** | **Total no of nuclei ^b^** | ***%*** (*p* = 0.25) |
| --- | --- | --- | --- | --- |
| Mll5+/+ | 4 | 161 | 81788 | 0.17 |
| Mll5 -/- | 4 | 313 | 55532 | 0.36 |

^a^ Apoptosis was determined using the TUNEL assay on paraffin section of testes.

^b^ An automated counter (Ariol Telepathology Software, Genetix Ltd, Hampshire, UK) was used to score the number of cells in each category. Four similar fields with seminiferous tubules were selected at random and the total number of nuclei and number of positive nuclei was added across these fields.
