# Supplementary material for: Mll5 Is Required for Normal Spermatogenesis
Source: PLoS One. 2011 Nov 1;6(11):e27127. doi: 10.1371/journal.pone.0027127 (PMC3206077; doi:10.1371/journal.pone.0027127)
Supplement: Data S1 — Supplemental data. (DOC) [file pone.0027127.s010.doc]

**Data S1** - Mll5 is required for normal spermatogenesis

**Female Mll5 -/- mice phenotype**

Wild type males with homozygous females were bred over a 4-month period. Table S1 shows that homozygous females mated with wild type males produced an average of 4.47 pups per pregnancy (76 pups/17 litters), a frequency not significantly different to wild type pairs that produced 4.82 pups per pregnancy (106 pups / 22 litters) (Pearson chi-square, p=0.62), demonstrating that homozygous female *Mll5^tm1Apa^* mice are fertile and able to bear live pups, with a frequency indistinguishable from wild type. Intriguingly, we noted a possible rearing deficit in homozygous mutant females, in that only 8 of 76 (10.5%) heterozygous pups survived to weaning whereas 96/106 (90.6%) pups from wild type littermates were alive at weaning (Fisher’s exact test, p <10^-15^). This difference in survival is unlikely to be due to genotype since 56% of pups weaned from heterozygous breeding pairs are heterozygous pups and 33% are wild type.

**Male Mll5 -/- secondary sexual characteristics**

Lack of virilising effects of testosterone can result in smaller testes and absent or diminished secondary sexual characteristics. We found no evidence of significant differences (p=0.96) in testis weight of Mll5 -/- males (Table S2). Moreover, homozygous male mice exhibit normal mating behavior and secondary characteristics such as developed preputial glands, making hormonal failure an unlikely cause of the infertility. Furthermore, testosterone levels in singly caged males of both wild type and homozygous animals show the expected variations in testosterone, but with similar mean values (p= 0.76, Table S3). These data are consistent with normal gonadotrophic stimulation of the gonads.

## Early gametogenesis appears to be intact in homozygous *Mll5 ^tm1Apa^ male mice*

Examination of hematoxylin and eosin (H&E) stained tissue sections from a total of 13 homozygous and 15 wild type testes showed no evidence of gross morphological differences between wild type and mutant animals. Specifically, all stages of spermatogenesis could be identified in both genotypes (Figure S1), and mature spermatozoa were found in the epididymides of both genotypes. Consistent with these findings, an assay of the apoptotic cells in the testis using a TUNEL assay (Table S4) showed a subtle but not statistically significant difference (Likelihood ratio χ^2^_df=1_ = 1.35, p=0.25) between the proportion of apoptotic cells in the seminiferous tubules in the testes of wild type (0.17% TUNEL positive nuclei) and homozygous *Mll5^tm1/Apa^* mice (0.36% TUNEL positive nuclei).
