# Supplementary material for: Mll5 Is Required for Normal Spermatogenesis
Source: PLoS One. 2011 Nov 1;6(11):e27127. doi: 10.1371/journal.pone.0027127 (PMC3206077; doi:10.1371/journal.pone.0027127)
Supplement: Methods S1 — Supplemental methods. (DOC) [file pone.0027127.s011.doc]

**Methods S1**

## Histology of mouse tissues

Briefly, mouse tissues were dissected from euthanized animals and fixed for four hours to overnight in 10% neutral buffered formalin solution, embedded in paraffin and sectioned at 4 to 7 µm. Tissue sections were stained with hematoxylin and eosin (H&E) or PAS (Perodic acid and Schiff’s Reagent which stains carbohydrates, glycogen and basement membranes magenta). Images were visualized using a Leica light microscope and 10x or 40x objectives. A Leica EC3 color camera was used to capture images.

**In situ hybridization (ISH)**

Testes from wild-type mice were snap frozen and sectioned to 10-15 microns on a microscope slide. Specifically, slides were heated at 50°C for three minutes and allowed to air dry at RT for 30 minutes. They were then treated with chloroform for 5 minutes and allowed to dry for 5 minutes. The slides were then treated with 4% paraformaldehyde in PBS for 7 minutes then washed in PBS, followed by washes in 2X SSC. The prehybridization step used 100 ul of hybridization buffer (4X SSC, 10% dextran sulfate, 1X Denhardt's solution (Invitrogen) 2mM EDTA, 50% deionized formamide, 500ug/ml salmon sperm DNA) at 42°C for 1 hour. The sense and antisense probes were added to the hybridization buffer at a concentration of 200ng/ul and slides were incubated with the probes in a humidified chamber overnight at 42°C.

The probes were created by PCR amplification using WT mouse testes cDNA and cloned into pSPT19 in both directions. The primer sequences were Mll5_F7 TCAGCACATCTGAAGATGGAA, Mll5_R7 CAGGAATATGCTGCCTGTCA. The PCR product produced a single band on gel electrophoresis, excised and cloned into pSPT-19 which was Sanger sequenced to confirm the sequence and orientation of the insert. The Roche Digoxigenin (DIG) RNA Labeling Kit was used to create the DIG label sense and antisense probes using T7 polymerase for both strands to generate the sense and antisense probes respectively as per manufacturer's recommendation.

After probe hybridization the slides were washed with 2X SSC followed by a 60% formamide in 0.2X SSC wash both at 37°C. There were final washes with 2X SSC at RT and with 100mM Tris-HCl, 150mM NaCl (pH 7.5) prior to probe detection. The Roche DIG Nucleic Acid Detection Kit (NBT/BCIP) was used to visualize the ISH probes. Specifically, the sections were incubated for 30 minutes at RT with blocking buffer (100mM Tris-HCl, 150 mM NaCl used to dilute 10X blocking reagent to 1X). The alkaline phospahatase-conjugated sheep anti-DIG was diluted to 1/200 in the blocking buffer and used to incubate the slides for 2 hours at RT. After incubation the slides were washed with 100mM Tris-HCl, 150 mM NaCl and incubated for 10 minutes with detection buffer (100nM Tris-HCl, 100mM NaCl, 50 mM MgCl_2_). Slides were incubated overnight with detection buffer containing 0.18 mg/ml 5-bromo-4-chloro-3-indolyl-phosphate, 0.34 mg/ml nitroblue tetrazolium chloride (Roche kit) and 240 ug/ml levamisole at RT. After a distilled water wash the slides were counterstained with 1% methylene green and mounted. Images were viewed using the 63x oil objective on a Zeiss Observer Z1 and captured on a Zeiss AxioCam MRc.

## Apoptosis analysis

The terminal deoxynucleotidyl transferase (TdT) dUTP nick end labeling (TUNEL) was performed using the ApopTag Plus Peroxidase In Situ Apoptosis Detection Kit following the manufacturer’s guidelines (Chemicon International). Basically, tissues were treated with Proteinase K and 3% hydrogen peroxidase before TdT enzyme was used to label DNA end breaks characteristic of apoptosis. This was visualized using anti-Digoxigenin-peroxidase and DAB before being mounted with a coverslip. The slides were then scanned and analyzed using the Ariol imaging software, where positive nuclei appeared dark brown.

## Microarray Analysis and Validation

Total RNA was isolated from three frozen wild type and age-matched homozygous *Mll5^tm1apa^* testes using QIAzol™ lysis reagent (Qiagen, Maryland, USA). The homogenized tissue in 800 µL QIAzol was transferred to Phase Lock Gel tubes (2 mL, heavy) (Eppendorf AG, Hamburg, Germany) to which 200 µL chloroform was added. Samples were centrifuged and the aqueous phase was removed and transferred to a new tube with an equal volume of isopropanol. After centrifugation, the supernatant was removed from the precipitated RNA pellets, and the pellets were washed with 70% ethanol. The air-dried RNA was dissolved in RNase/DNase free water and stored frozen at -80 °C.

Total RNA samples were analyzed on a NanoDrop (Thermo Fisher Scientific, Waltham, MA) spectrophotometer to determine RNA quality and concentration. One µL of the RNA (was adjusted within a range of 25-500 ng/µL) was analyzed on the Agilent 2100 Bioanalyzer (Agilent Technologies, Waldbronn, Germany) using the RNA 6000 Nano kit. Samples with a resulting total RNA of 1 µg or greater and with a RNA integrity number (RIN) of 7.0 or greater were probed on GeneChip Mouse Exon Array 1.0 ST chips (Affymetrix, Santa Clara, California) for gene expression analysis.

The whole transcript sense target labeling procedure followed the Affymetrix protocol, including the RiboMinus™ rRNA removal step. Three biological replicates were compared for each of the genotypes (wild type and homozygous *Mll5^tm1Apa^*) for a total of six experiments. Raw intensity calls were normalized using quantile normalization [40] and probeset summarization undertaken with gc-rma [41]. In this analysis we considered only transcript level summarization for the core plus extended set of probes on the array (about 800,000 probesets). The ratio of expression between wild type and mutant transcriptomes is summarized in Figure S2. Of note, the array hybridization was sensitive enough to unambiguously detect the specific 180 bp deletion in *Mll5* exon 3 of the *mll5^tm1Apa^* allele in the exon hybridisation. Outliers with greater than 1.5 fold change between genotypes and level of significance, p < 0.05 were shortlisted selected for further analysis by quantitative PCR (Q-PCR) (Figure S3). We validated 26 transcripts by Q-PCR (Figure S4) on an additional cohort of three wild type and three homozygous *Mll5^tm1apa^* testes using *Eef1a1* as an endogenous control for RNA loading. Two to four technical replicates per biological sample were measured. Since we did not know the function of Mll5 and how it might affect common house-keeping genes, we obtained the endogenous control from the microarray data as the gene which showed the least variability and differential expression between wild type and homozygous *Mll5^tm1Apa^* testes samples.

For Q-PCR, the Invitrogen first stand synthesis kit was used to convert 2 µg of input total RNA into cDNA. Quantitative real-time polymerase chain reaction (Q-PCR) was set up in 384 well plates on the 7900HT Fast Real-Time PCR system (Applied Biosystems, Foster City, CA) in a reaction volume of 10µl per well each containing 400 nM of forward and reverse primers, 100 nM of the respective Roche UPL probe and 1X Applied Biosystems (ABI) master mix (with Rox as a loading control) containing 10 ng of template cDNA. Specific probes for each transcript were identified with matching primers using the online tool at UPL Assay Design Center (Roche Applied Science). For transcripts without a corresponding probe, we used the SYBR green master mix (Roche). The probes were obtained from the UPL (Roche) and primers synthesized by Invitrogen for each gene and are listed in Figure S4. Q-PCR C_t_ data was modeled using a linear mixed effects model with genotype and gene target as fixed effects and biological replicates as a random effect. The transcript *Eef1a1* was used as an endogenous loading control. Relative quantification (RQ) of the mutants compared to wild type was determined as 2^‑∆∆C^_t_, with ∆∆C_t_ obtained from the parameters of the linear mixed effects model. Tests for genotype effect on gene of interest adjusted for amplification using the endogenous control were carried out using the likelihood ratio test.
